# Radiographic characteristics in congenital scoliosis associated with split cord malformation: a retrospective study of 266 surgical cases

**DOI:** 10.1186/s12891-017-1782-z

**Published:** 2017-10-23

**Authors:** Fan Feng, Haining Tan, Xingye Li, Chong Chen, Zheng Li, Jianguo Zhang, Jianxiong Shen

**Affiliations:** 1 Department of Orthopedics, Peking Union Medical College Hospital, Chinese Academy of Medical Science, Peking Union Medical College, Beijing, China; 2 Department of Orthopedic Surgery, Peking Union Medical College Hospital, Chinese Academy of Medical Science, Peking Union Medical College, No. 1 Shuai Fu Yuan, Wang Fu Jing Street, Beijing, 100730 China

**Keywords:** Congenital scoliosis, Split cord malformation, Intraspinal anomalies, Deformity

## Abstract

**Background:**

Vertebrae, ribs, and spinal cord are anatomically adjacent structures, and their close relationships are clinically important for planning better corrective surgical approach. The objective is to identify the radiographic characteristics in surgical patients with congenital scoliosis (CS) and coexisting split cord malformation (SCM).

**Methods:**

A total of 266 patients with CS and SCM underwent surgical treatment at our hospital between May 2000 and December 2015 was retrospectively identified. The demographic distribution and radiographic data were collected to investigate the characteristics of spine curve, vertebral, rib, and intraspinal anomalies. According to Pang’s classification, all patients were divided into two groups: type I group is defined as two hemicords, each within a separate dural tube separated by a bony or cartilaginous medial spur, while type II group is defined as two hemicords within a single dural tube separated by a nonrigid fibrous septum.

**Results:**

There were 104 patients (39.1%) in Type I group and 162 patients (60.9%) in Type II group. SCM was most commonly found in the lower thoracic and lumbar regions. The mean length of the septum in Type I SCM was significantly shorter than Type II SCM (2.7 vs. 5.2 segments). Patients in Type I group had a higher proportion of kyphotic deformity (22.1%). The vertebral deformities were simple in only 16.5% and multiple in 83.5% of 266 cases. Patients in Type I group presented higher prevalence of multiple (90.4%) and extensive (5.1 segments) malformation of vertebrae. In addition, hypertrophic lamina and bulbous spinous processes were more frequent in Type I group (29.7%), even developing into the “volcano-shape” deformities. Rib anomalies occurred in 62.8% of all patients and 46.1% of them were complex anomalies. The overall prevalence of other intraspinal anomalies was 42.9%. The most common coexisting intraspinal anomalies was syringomyelia (30.5%).

**Conclusion:**

The current study, with the largest cohort to date, demonstrated that patients with CS and coexisting SCM presented high prevalence of multiple vertebral deformities, rib and intraspinal anomalies. The length of the split segment in Type I SCM was shorter than that in Type II SCM. Compared with Type II SCM, patients with Type I SCM presented with higher incidence of kyphotic deformity, more extensive and complicated vertebral anomalies, and more complex rib anomalies.

## Background

Congenial scoliosis (CS) is a kind of rare spinal deformity. Large population studies utilizing screening low-dose radiography of the spine suggested a congenital scoliosis prevalence of 0.5% to 0.11% [1, 2]. CS identified at birth that is a byproduct of anomalous vertebral development in the embryo (the first 8 weeks of gestation) [3]. During this period, the bony elements of the spine are forming, and the neuraxis is completing its infolding, closing the neural tube [4]. These events are closely related, and any intrauterine event that causes CS could also be associated with an occult intraspinal anomalies (incidence ranged from 18% to 38%) [5–7]. These anomalies include syringomyelia, tethering of the cord, low conus, lipoma, but the most common one is split cord malformation (SCM) [3, 7, 8].

SCM is a relatively rare form of a congenital neural anomaly and tethered spinal cord syndrome. According to Pang’s classification [9, 10], type I SCM is defined as two hemicords, each within a separate dural tube separated by a bony or cartilaginous medial spur, while type II SCM is defined as two hemicords within a single dural tube separated by a nonrigid fibrous septum. Both these conditions may limit movement of the spinal cord within the canal, possibly placing the patient at an increased neurological risk when performing surgical treatment for congenital scoliosis [11–14].

Previous studies had revealed the close relationships between rib anomalies and vertebral or intraspinal anomalies [15–17]. Vertebrae, ribs, and spinal cord are anatomically adjacent structures, and a local developmental deficiency in this area may explain the synchronous occurrence of these anomalies [18]. These various anomalies are substantial clues for better identifying of the scoliotic characteristics, and at the same time clinically important for planning better corrective surgical approach.

However, to the best of our knowledge, there have been no studies that have specifically investigated the radiographic details of CS patients associated with SCM. Therefore, the purpose of this study is to identify the vertebral, rib and intraspinal anomalies in surgical patients with congenital scoliosis and coexisting split cord malformation. Based upon these findings, it could potentially be useful for improving surgical strategies in these individuals.

## Methods

After obtaining approval from the institutional review board in our hospital, 281 consecutive patients of congenital scoliosis associated with SCM undergoing spinal correction surgery between May 2000 and December 2015 were retrospectively reviewed. Among these patients, 266 fulfilled the following inclusion criteria: (1) definite diagnosis of congenital scoliosis with SCM; (2) patients with complete medical records and radiological data. 21 Patients with previous spinal surgery, spinal fractures were excluded. Each patient received examinations of an entire spine magnetic resonance imaging (MRI) and computerized tomography (CT) to evaluate the type and location of SCM. According to Pang’s classification for SCM [9, 10], all patients were divided into Type I group and Type II group.

### Radiographical measurements

The standing posterior-anterior radiographs were reviewed to identify the type of the curvature according to the classification proposed by the Scoliosis Research Society [19]: thoracic (apex between T2 and T11), thoracolumbar (apex between T12 and L1), lumbar (apex between L2 and L4). The patients with kyphotic deformities on the standing lateral radiographs were also recorded. Congenital kyphosis is defined as a sagittal plane deformity: thoracic region (T5-T12) > 40° or thoracolumbar region (T10-L2) >30° or kyphosis at lumbar region (L1-L5) or angular kyphosis at any segments of spine [20].

The plain radiograph and 3-dimensional reconstruction CT of the entire spine were reviewed to evaluate the presence of vertebral deformities as well as rib anomalies. The vertebral anomalies and the type of spine deformity were categorized according to the classification proposed by Winter et al. [21, 22] and McMaster et al. [23] into failures of vertebral segmentation, failures of vertebral formation, and mixed anomalies. Furthermore, vertebral anomalies were classified as single malformations (failure of formation of one vertebra or failure of segmentation of the adjacent two vertebrae) or multiple malformations (failure of segmentation of more than two vertebrae, or mixed anomalies of multiple vertebras). Their number and site of the vertebral deformities were also recorded.

Rib anomalies which included number changes (increased or decreased ribs) and structural deformities (fused, bifid, and widened/irregular ribs), can be classified as simple or complex type [15]. Patients with simple type of rib anomaly were defined as a localized fusion of two or three ribs or a small chest wall defect resulting from an anomalies of one or two ribs or an absence of a rib. Patients with complex type of rib anomalies were described as multiple extensive rib fusions, usually without a fixed pattern, association with a large chest wall defect due to an absence or variation of ribs. The side and location of rib anomalies were recorded as well, including upper thoracic (T1–T4), middle thoracic (T5–T8), or lower thoracic (T9–T12).

### Data collection

All of the measurements were performed by an independent observer. Each parameter was measured for 2 times, and the average values were calculated in both groups.

### Statistics

The data were analyzed using SPSS version 17.0 for Windows (SPSS Inc., Chicago, IL).The independent sample *t* test and Pearson Chi-square test was performed to analyze the differences between the 2 groups where appropriate. Statistical significance was considered with a *P* value <0.05.

## Results

**Fig. 1:**
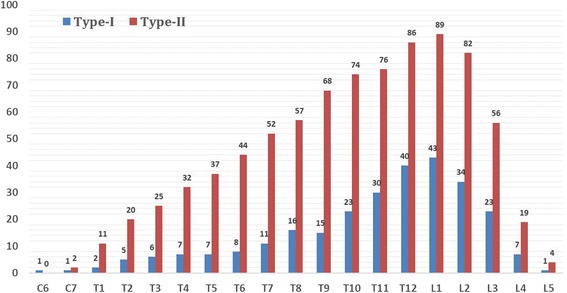
The location details of septum in two types of split cord malformation

The Type I group consisted of 76 female and 28 males (38.2%) with an average age of 14.0 years (range, 4–39 yr); while the Type II group consisted of 114 female and 48 males (61.8%) with a mean age of 14.3 years (range, 3–38 yr). No significant differences were detected between two groups in gender (*P* = 0.633) or age (*P* = 0.715). Most of the split were located at the lower thoracic and lumbar regions. Location details are shown in Fig. 1. The length of the split segment was measured according to the number of vertebral bodies it spanned. In Type I group, the length of the split segment extended with an average of 2.7 (range, 1–8) segments. While in Type II group, it varied between 1and 16 segments, with a mean of 5.2 segments. This difference was statistically significant (*P* < 0.001) (Table 1).

**Table 1:** Radiographic characteristics of patients with congenital scoliosis and SCM

characteristics	Type I	Type II	P
Number of patient, n (%)	104 (39.1)	162 (60.9)	
Age (yrs)	14.0 ± 6.0	14.3 ± 5.9	0.715
Sex (M/F)	28/76	48/114	0.633
Length of split segment, n	2.7 ± 1.5	5.2 ± 3.3	< 0.001*
Main curve Cobb angle (°)	71.5 ± 27.4	68.9 ± 24.1	0.434
Type of curve, n (%)			0.448
Thoracic	67 (64.4)	112 (69.1)	
Thoracolumbar	23 (22.1)	36 (22.2)	
Lumbar	14 (13.5)	14 (8.6)	
Kyphotic deformity			0.029 *
With, n (%)	25 (24.0)	22 (13.6)	
Without	79	140	

*SCM* split cord malformation; * means statistical difference

**Fig. 2:**
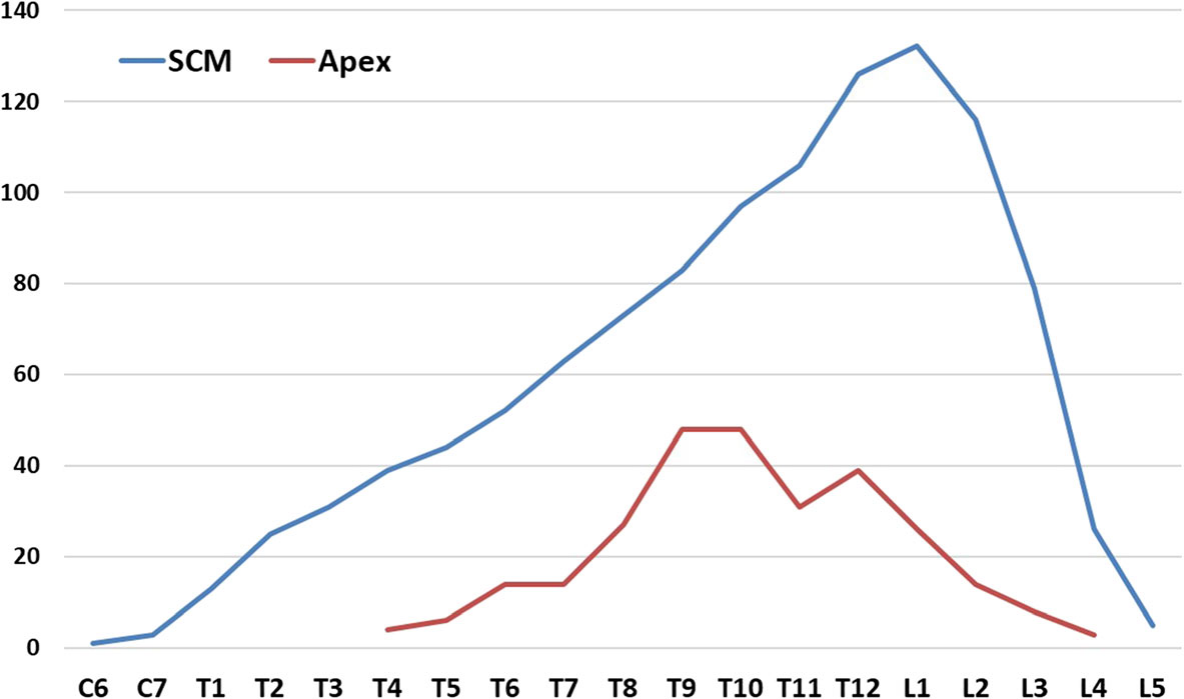
The location distribution of the septum (one peak) and the apex of scoliosis (two peaks)

The average Cobb angle of the main curve was 71.5° (range 14°–165°) in Type I group and 68.9° (range 30°–158°) in Type II group. There were no significant differences between two groups (*P =* 0.434). Thoracic curve was the most common curve pattern (179, 67.3%), followed by thoracolumbar (59, 22.2%) and lumbar (28, 10.5%). Though the most common location of the septum located at thoracolumbar region, while the apex of major curve mostly located at the middle thoracic and thoracolumbar regions (Fig. 2). Moreover, kyphotic deformity was found to be more frequent in Type I group (25, 24.0%) than in Type II group (22, 13.6%) (χ^2^ = 4.762, *P =* 0.029) (Table 1).

### Vertebral anomalies

A total of 44 cases (16.5%) presented with single malformation of vertebral anomalies, whereas the other 222 cases (83.5%) had multiple malformations. Failure in segmentation, formation, and mixed anomalies was observed in 28.6%, 16.9%, and 54.5% of cases, respectively. Compared with Type II group, the patients of Type I group presented higher prevalence of multiple malformations and mixed anomalies. Besides, the involved levels of vertebral deformities was also longer (5.1 vs. 3.9, *P* < 0.001). The vertebral anomalies in Type I group were most frequently found in both thoracic and lumbar regions, whereas the vertebral anomalies in Type II group were most frequently found only in thoracic region (*P* = 0.004).

**Fig. 3:**
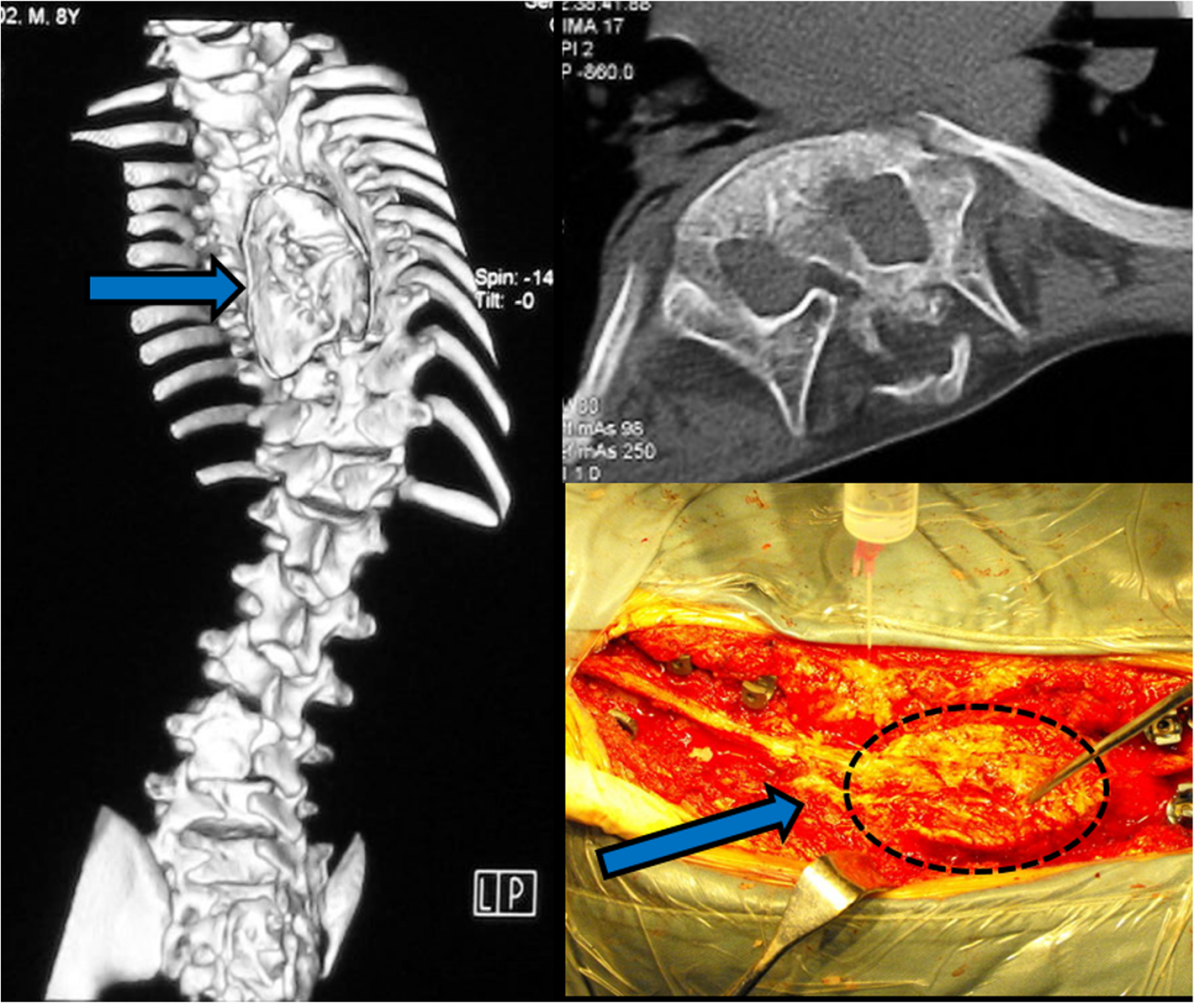
Images of an eight-year-old male patient diagnosed with congenital scoliosis and the type I split cord malformation. Three-dimension CT showed hypertrophic lamina and bulbous spinous processes in the middle thoracic region. These exuberant neural arches can be described as the “volcano-shape” deformity (with arrows in the figure)

Of note, hypertrophic lamina and bulbous spinous processes were present in 32 (30.8%) patients with Type I SCM. Occasionally, three or more adjacent lamina were fused into a massive gnarl, which we described as the “volcano-shape” deformity (Fig. 3) [10]. In contrast, the exuberant neural arches were observed in only 21 cases (13.0%) of Type II group. There were significant differences between two groups (χ^2^ = 12.587, *P <* 0.001) (Table 2).

**Table 2:** Vertebral anomalies in patients with congenital scoliosis and SCM

characteristics	Type I (*n* = 104)	Type II (*n* = 162)	P
Vertebral deformities, n (%)			0.001*
Failure of segmentation	21 (20.2)	55 (34.0)	
Failure of formation	11 (10.6)	34 (21.0)	
Mixed type	72 (69.2)	73 (45.1)	
Malformation, n (%)			0.015*
Single malformation	10 (9.6)	34 (21.0)	
Multiple malformation	94 (90.4)	128 (79.0)	
Levels of vertebral deformities, n	5.1 ± 2.5	3.9 ± 2.2	< 0.001*
Location of deformities, n (%)			0.004*
T	65 (62.5)	126 (77.8)	
L	4 (3.8)	10 (6.2)	
T + L	35 (33.7)	26 (16.0)	
Exuberant neural arches, n (%)			< 0.001*
with	32 (30.8)	21 (13.0)	
without	72 (69.2)	141 (87.0)	

*SCM* split cord malformation; * means statistical difference

### Rib anomalies

Rib anomalies occurred in 62.8% (167/266) of the patients. Of these, 90 (53.9%) had simple anomalies and 77 (46.1%) had complex anomalies. Compared with Type II group, the patients of Type I group presented higher prevalence of complex rib anomalies (29.7% vs. 47.0%, *P =* 0.028). Rib anomalies occurred mostly on the concavity of the scoliosis in 65.9% of cases (110/167), followed by bilateral (22.8%) and the convexity (11.4%). There were no significant differences in the type and distribution of rib anomalies between two groups (Table 3). With regard to the rib structural changes, the most common was fused ribs (50.3%), followed by bifid rib combined with fused ribs (21.0%) and bifid ribs (12.7%).

**Table 3:** Rib anomalies in patients with congenital scoliosis and SCM

characteristics	Type I (n = 104)	Type II (n = 162)	P
Rib anomalies, n (%)			0.854
without	38 (36.5)	61 (37.7)	
with	66 (63.5)	101 (62.3)	
Type, n (%)			0.023*
simple	35 (53.0)	71 (70.3)	
complex	31 (47.0)	30 (29.7)	
Side of scoliosis, n (%)			0.216
concave	46 (69.7)	64 (63.3)	
convex	4 (6.1)	15 (14.9)	
bilateral	16 (24.2)	22 (21.8)	
Type of structural changes, n (%)			0.540
fused ribs	32 (48.5)	52 (51.5)	
bifid ribs	5 (7.6)	15 (14.9)	
bifid rib combined with fused ribs	13 (19.7)	22 (21.8)	

*SCM* split cord malformation; * means statistical difference

### Other intraspinal abnormalities

In this study, a total of 114 (42.9%) patients were found to have other intraspinal anomalies. The most common anomaly was syringomyelia (81, 30.5%), followed by low conus (67, 25.2%) and tethered cord (60, 22.6%). 29 patients (10.9%, 15 with Type I SCM and 14 with Type II SCM) had concurrent anomalies of SCM, syringomyelia, and tethered cord. No significant differences were observed in the distribution between two groups. (Table 4).

**Table 4:** Other intraspinal abnormalities in patients with congenital scoliosis and SCM

characteristics	Type I (n = 104)	Type II (n = 162)	P
With other intraspinal abnormalities, n (%)	46 (44.2)	68 (42.0)	0.717
Syringomyelia	32 (30.8)	49 (30.2)	0.928
Tethered cord	27 (26.0)	33 (20.4)	0.287
Low conus	30 (28.8)	37 (22.8)	0.271
Intraspinal masses	3 (2.9)	4 (2.5)	0.836

*SCM* split cord malformation

## Discussion

The present study is the first investigation with large cohort in the literature that examines vertebral, rib, and intraspinal anomalies at the same time in patients with CS and coexisting SCM. In our study of 266 CS patients, patients with fibrous septum were nearly 1.5 times those with bony septum, which was higher than the results of previous studies [10, 24]. Regardless of the type, the lower thoracic and lumbar regions were the most common site for SCM. No differences were detected in the distribution between two types of SCM. This conformity could be explained by Pang’s theory for the formation of SCM, a common origin of both malformation that an adhesion between the ectoderm and endoderm—the accessory neurenteric canal—leads to an endomesenchymal tract which bisects the spinal cord [9]. Pang et al. [10] also showed that he split lengths of the 19 Type I lesions (2.97 levels) were longer than that of 18 Type II lesions (1.17 levels). However, in our series, the length of the split segment in both group were both longer and the septum in Type I group was significantly shorter than that in Type II group (2.7 vs. 5.2). Therefore, there are still reasons to concern about the neurological risk due to the tethering function of the septum, anchoring the spinal hemicords.

Ghandhari et al. [17] reported 202 CS patients, in which vertebral defects were failure in formation (53.5%), failure in segmentation (31.7%), and a mixed form of both (14.9%). Their rates are in conformity with previous reports for common CS patients [16, 25]. However, in our series, failure in segmentation, formation, and mixed type was observed in 28.6%, 16.9%, and 54.5% of 266 cases. The prevalence of mixed type was much higher than those reported in common CS patients. In fact, this result is in line with previous studies that intraspinal anomaly were more common in patients with congenital scoliosis resulting from mixed defects [3, 8]. Therefore, single malformation composed of only 16.5% of vertebral deformities, while 83.5% of cases had multiple malformations. Furthermore, when compared with Type II group, the patients in Type I group presented higher prevalence of multiple malformations and mixed anomalies of vertebral deformities. And the length of involved levels is also larger. These findings may have some important implications for the pedicle screw–based correction of CS with SCM. Firstly, the vertebral deformities of Type I SCM patients tend to more complex. These severe and irregular deformities make the spinal anatomic landmark obscure, which may increase the possibility of malpositioned pedicle screws. Secondly, multiple malformations and mixed anomalies of vertebral deformities, as well as the length of involved levels, would severely decrease the flexibility of spine. In contrast, the Type II SCM always associated with simple vertebral abnormality, thus it is more likely to get better curve correction. Consequently, it is not hard to understand the results of previous studies [12, 13] that the correction rate of Type II is higher than that of Type I SCM. The spine surgeon should have a clear that the most important issue for these patients is to arrest the progression of scoliosis and deterioration of neurological deficits, rather than just pursuing overcorrection of spinal deformity.

Notably, hypertrophic lamina and bulbous spinous processes, even developing into the “volcano-shape” deformity (Fig. 3), were present in 32 (30.8%) patients with Type I SCM. In contrast, these exuberant neural arches were seen in only 13.0% Type II cases. In Pang’s series [10], similar findings were found in 16 of 19 Type I cases and 2 of the 18 Type II cases. Traditionally, to avoid creating a neural deficit or aggravating a preexisting neural deficit, resection of the septum has been indicated for patients with SCM prior to any procedure for correcting the spinal deformity [10, 24, 26–28]. However, in case with “volcano-shape” deformity, it is technically difficult to remove the posterior elements at the level of septum during the prophylactic neurosurgery.

Xue et al. [16] reported that rib anomalies were present in 50.3% of Chinese patients with CS. Similarly, the prevalence of rib anomalies was 57.4% in another report by Ghandhari et al. [17]. However, in this study, we found a considerably higher prevalence (62.8%) of rib anomalies among CS patients with SCM. This difference is easy to understand because prior studies have suggested a correlation between rib anomalies and occult intraspinal anomaly (SCM is the most common intraspinal anomaly) [15–17]. The prevalence of intraspinal anomaly was significant higher in the patients with rib anomalies than those without rib anomalies. Thus, as expected, a greater number of rib anomalies can be identified in CS patients with SCM. Furthermore, rib anomalies in the present study consisted of simple anomalies (53.9%) and complex anomalies (46.1%). In contrast with previous reports [15, 17] (21.0~29.1%), the rate of complex rib anomalies was relatively higher, especially in Type I SCM patients. Clinically, these rib anomalies could significantly compromise the correction rate in congenital scoliosis [29].

In a Chinese series [8], the prevalence of syringomyelia, low conus and tethered cord in CS patients were only 18.1%, 5.8% and 11.5%, respectively. Ghandhari et al. [17] reported the intraspinal anomalies were found in 21.8% of patients, including syringomyelia (9.9%), tethered cord (3%), low conus (3%), and intraspinal tumors (2%). In contrast, we firstly revealed the high prevalence (42.9%) of intraspinal anomalies in the largest sample of CS patients with SCM. Syringomyelia was detected most frequently (30.5%), followed by low conus (25.2%) and tethered cord (22.6%). These findings of multiple intraspinal anomalies may be explained by the important embryological mechanisms: the codetermination in the embryonic developmental time of intraspinal anomalies and congenital spinal deformity [3, 30]. On the other hand, all patients in the current study underwent a full spine MRI. Some earlier studies of the instance of intraspinal anomalies have not demanded this, and as a result, the instance of syringomyelia may be understated, as the syringomyelia has been missed. More importantly, spine surgeons should have a clear knowledge that patients having CS and SCM may also closely associated with other intraspinal anomalies. These coexisting tethering lesions, especially the tethered cord, is stretch-induced functional disorder of the spinal cord and anchors the caudal end of the spinal cord which prevents cephalad movement of the lumbosacral cord [31]. In our experience, it should be prudent to perform prophylactic neurosurgery of spur excision and cord untethering before corrective surgery. Some prior studies had revealed that excision of the spur did not result in a dramatic improvement in the neurological status [24, 32, 33]. For these patients, their neurological deficits may result from other intraspinal anomalies, rather than the only tethering dysfunction of SCM itself.

Two limitations to this study should be addressed. First, this is a retrospective study based on operated cases to demonstrate the radiological features of patients with CS and SCM, which might be helpful to planning better treatment. However, it should be acknowledged that in patients not being operated, the prevalence or extent of anomalies might be different. Second, more basic researches focusing on the pathogenesis of CS and coexisting SCM are warranted to reveal the mechanism of these finding in current study.

## Conclusions

The results of our study, for the first time with the largest sample size, demonstrated the radiographic characteristics of CS patients with coexisting SCM. Patients with coexisting CS and SCM present relatively high incidence of multiple vertebral deformities, rib and intraspinal anomalies. The length of the split segment in Type I SCM was significantly shorter than that in Type II SCM. Compared with Type II SCM, patients with Type I SCM presented with higher incidence of kyphotic deformity, more extensive and complicated vertebral anomalies, and more complex rib anomalies. These findings may provide important clues for better understanding their clinical features and thus improving the safety and efficacy of surgical treatment for patients with congenital scoliosis and coexisting split cord malformation.

## Abbreviations

CS: congenital scoliosis
SCM: split cord malformation
